# Agricultural visual recognition: a novel privacy-preserving framework based on 4D chaos and Mamba networks

**DOI:** 10.3389/fpls.2026.1939691

**Published:** 2026-09-30

**Authors:** Hai Yu, Yutao Xia, Yandong Ding, Xin Li, Zhenqi Cheng, Jiao Han

**Affiliations:** Weifang University of Science Technology, Weifang, China

**Keywords:** 4D chaotic system, crop disease detection, Mamba-LSDet, smart agriculture, visual recognition

## Abstract

With the advancement of smart agriculture and Internet of Things (IoT) based monitoring systems, secure and accurate crop disease recognition for large-scale agricultural management remains challenging. Existing methods mainly focus on recognition performance while insufficiently considering data privacy protection and computational efficiency, which limits their deployment in resource-constrained agricultural environments. To address these issues, this study proposes a privacy-preserving crop disease recognition framework that combines multi-image encryption and lightweight detection. First, a four-dimensional chaotic encryption scheme is developed to secure agricultural image transmission by incorporating chaotic sequence generation, multi-channel pixel permutation, and DNA-based diffusion operations. The proposed encryption method improves data security and anti-attack capability while maintaining low computational cost. Furthermore, a lightweight Mamba-LSDet network is designed for crop disease recognition after secure image transmission and decryption. Equipped with a cross-stage gated module for adaptive feature extraction and a shared prediction head for parameter reduction, the network achieves accurate and efficient disease detection. Experimental evaluations demonstrate the effectiveness of the proposed encryption scheme in terms of security and efficiency. Moreover, detection experiments after image decryption show that Mamba-LSDet achieves mAP@50 and mAP@50–95 values of 0.841 and 0.505, respectively, outperforming the lightweight YOLOv11n model. The proposed framework provides an effective solution for secure and lightweight crop disease recognition in agricultural IoT applications.

## 1 Introduction

Large-scale distributed sensing networks have become fundamental for data collection, transmission and analysis in modern intelligent systems. Within Agricultural Internet of Things (Ag-IoT) scenarios, distributed cameras Barjaktarovic et al. (2024), environmental sensors and embedded terminals jointly produce multi-type data streams to facilitate agricultural intelligent decision-making. Visual data stands out among all modalities, since image analytics support fine-grained agricultural tasks including crop disease identification and growth status evaluation Ngugi et al. (2024). For visual crop disease recognition, accurate interpretation heavily depends on the confidentiality and integrity of transmitted agricultural images.

The distributed and resource-constrained nature of Ag-IoT networks presents substantial challenges to secure information processing. As agricultural production increasingly depends on data-driven decision making, the security of visual information becomes critical to both food safety and sustainable farming. Visual data transmitted over open wireless channels are vulnerable to interception, tampering, and unauthorized inference, which may cause privacy leakage and information loss. Agricultural image data face similar threats during transmission and storage Li et al. (2023). These security risks not only undermine data confidentiality but also degrade recognition accuracy and overall system reliability Ghosh et al. (2024). Consequently, secure transmission and intelligent recognition should be considered jointly as interdependent components of a unified information processing pipeline. Effective encryption mechanisms must protect sensitive visual content while retaining the semantic features required for downstream inference, and remain lightweight to meet stringent computational and energy constraints Wang et al. (2024); Zuhair et al. (2023).

Although existing studies have achieved considerable progress in image encryption and crop disease recognition, these two aspects are generally investigated independently. Encryption-oriented methods mainly emphasize security performance, such as randomness and resistance against attacks, but rarely consider the requirements of subsequent visual analysis. Meanwhile, lightweight detection models focus on improving recognition efficiency but usually assume that image data are directly available during transmission and inference, which may introduce privacy risks in agricultural IoT environments. Therefore, a unified framework that simultaneously ensures image confidentiality, computational efficiency, and accurate disease recognition is still required.

To this end, a privacy-preserving recognition framework is developed. Several recent studies have explored unified frameworks that integrate privacy-preserving mechanisms into visual sensing applications, such as agricultural disease detection Sangaiah et al. (2024) and healthcare image recognition Baranwal et al. (2024); Rehman et al. (2022) However, these studies mainly focus on single-image processing and offer limited applicability and practicality in multi-image scenarios. To address this limitation, a joint multi-image encryption scheme is developed. The proposed method uses a 256-bit key generated by SHA-256 to initialize a four-dimensional logistic–sine chaotic system. The resulting pseudo-random sequence drives pixel permutation, DNA-based diffusion, and multi-channel XOR operations, which effectively reduce spatial redundancy and statistical correlations among images. By embedding encryption into the data acquisition and transmission stages, the method ensures data confidentiality and reversibility while maintaining low computational overhead, making it suitable for edge-level intelligent systems.

After secure transmission and decryption, the recovered images are directly used for disease recognition. Although structured state space models (SSMs), such as Mamba Zhang et al. (2024b) and its variants Liu et al. (2025); Zhang et al. (2025), excel at modeling long-range dependencies, their causal sequence modeling is less suitable for static visual tasks. Inspired by MambaOut Yu and Wang (2024), a lightweight recognition network based on gated convolutional structures with global context modeling and multi-scale feature fusion, enabling accurate disease inference under limited computational resources. In summary, this paper presents an integrated information processing framework that unifies secure image transmission and intelligent disease recognition within a single system. By jointly considering information protection and semantic interpretation, the proposed approach forms a coherent pipeline from encrypted data acquisition to reliable inference. **Figure 1** illustrates the overall workflow of the proposed system. The main contributions of this paper are summarized as follows:

**Figure 1:**
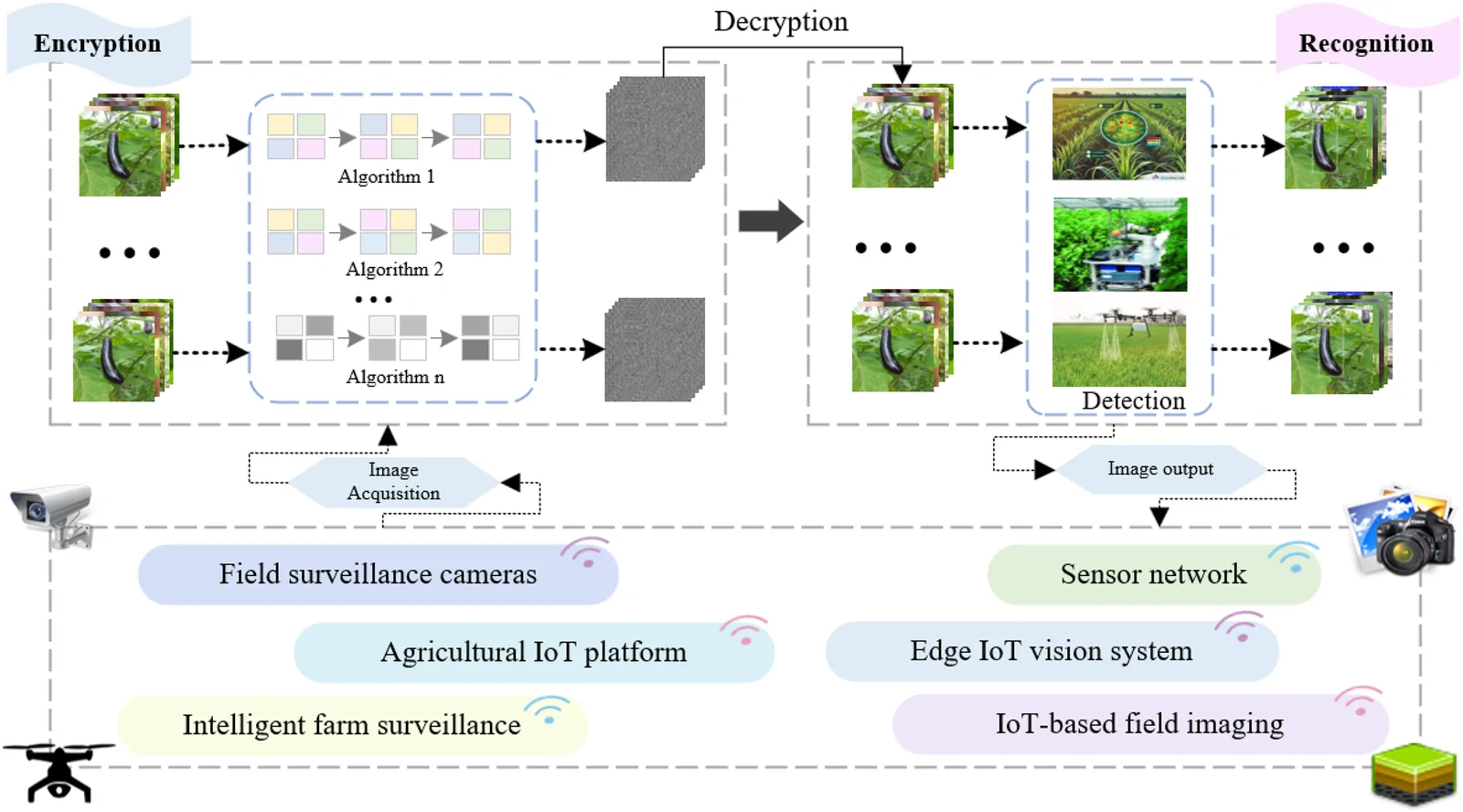
System flowchart.

- A framework that combines image encryption with a lightweight detection model is proposed to simultaneously ensure data security and detection accuracy. This framework enables integrated secure transmission and disease identification.
- A multi-image encryption scheme based on a four-dimensional chaotic system and DNA encoding is proposed. By combining key-controlled permutation, bit-level diffusion, and a global chain mechanism, the scheme achieves high encryption efficiency,strong security, and robust resistance to differential attacks.
- A lightweight detection network, Mamba-LSDet, is developed, which introduces a cross-stage gated module for dynamic feature selection and an efficient shared convolutional detection head to reduce redundant computation. Combined with a distribution-based loss, the model enhances fine-grained lesion representation while reducing parameters and GFLOPs.
- The proposed Mamba-LSDet achieves improved detection performance compared with the baseline YOLOv11n. On the eggplant disease dataset, the proposed approach improves mAP@50 from 0.825to 0.841 and mAP@50–95 from 0.488 to 0.505. It also achieveshigher precision and recall, with notable gains in key categories such as Fruit_Rot and Healthy.

The rest of this paper is organized as follows: Section 2 reviews related work. Section 3 introduces the proposed multi-image encryption scheme. Section 4 presents the Mamba-based lightweight shared detection network in detail. Section ref4 reports the experimental results and analysis of the integrated encryption and detection framework. Finally, Section 6 concludes the paper. Section 7 highlights the system’s significance, practical value, and limitations.

## 2 Related work

This section reviews related work on multi-image encryption and object detection, outlining the evolution from traditional methods to advanced approaches and highlighting recent efforts to integrate encryption with detection frameworks.

### 2.1 Multi-image encryption

With the rapid development of the Internet of Things and smart agriculture technologies, massive image data must be transmitted and stored over untrusted networks. Consequently, image encryption methods that balance security and efficiency have become a research focus. Early studies mainly targeted single-image encryption, employing pixel permutation and diffusion Moreira Bezerra et al. (2021), row–column transformation Zhao et al. (2024), and secure data transmission Jammula et al. (2022), typically relying on one- or two-dimensional chaotic systems for key sequence generation. For instance, Wang et al. Wang and Liu (2022) developed a one-dimensional dual-parameter hybrid coupled map lattice model, which uses edge detection to extract key features and employs three-dimensional bit-level XOR and dynamic exponential diffusion for encryption. Gao et al. (2025) proposed a parallel color image encryption scheme based on a two-dimensional logistic–Rulkov neuron map, which enhances randomness and complexity by combining logistic and Rulkov maps and improves efficiency through block-parallel processing. However, these approaches often suffer from high computational overhead and limited key sensitivity when handling large-scale data.

To overcome these limitations, researchers have proposed multi-image joint encryption schemes Gao and Teng (2025) that support simultaneous encryption through multi-channel fusion and multi-key collaborative control. Zhou et al. Zhou et al. (2023) designed a multi-channel encryption system integrating multiple optical components, achieving five independent information channels and significantly improving storage security and capacity. Qian et al. Qian et al. (2023) constructed a two-dimensional memristor-enhanced polynomial hyperchaotic map to design a highly secure and efficient multi-channel encryption algorithm. Liang et al. Liang et al. (2024) proposed a multi-key, multi-channel scheme based on a four-channel metasurface, enabling the integration of encryption and steganography. Moreover, the introduction of DNA coding.Goudjil et al. (2025) further improves randomness and security, while compressed sensing–based integrated approaches Yang et al. (2024) have also gained increasing attention. Despite theseadvancements, most existing schemes still face challenges such as high computational complexity, complicated implementation structures, and limited adaptability to resource-constrained agricultural IoT environments. Therefore, a lightweight multi-image encryption scheme with strong diffusion capability, efficient decryption, and practical deployment potential is still required. In this study, a four-dimensional chaotic encryption framework combined with DNA-based global diffusion is proposed to address these limitations while achieving a balance between security and computational efficiency.

### 2.2 Object detection

With the rapid development of deep learning, convolutional neural networks (CNNs) Iftikhar et al. (2024) have become the mainstream approach for crop disease detection. Early object detection methods, such as Faster R-CNN Du et al. (2022) and SSD Wenjuan et al. (2024), achieved high accuracy on large-scale datasets. For example, Li et al. Li et al. (2024) proposed a lightweight locality-aware Faster R-CNN for efficient pest detection and real-time monitoring. However, their large parameter size and slow inference limit deployment on edge devices.

To address these issues, lightweight detection networks such as MobileNet Quach et al. (2024) and ShuffleNet Yang et al. (2023) reduce computational cost through depthwise separable convolution and feature pyramid structures. Attention mechanisms, feature re-weighting, and multi-scale fusion further improve model robustness and feature representation. For instance, Zhou et al. Zhou et al. (2025) proposed LN-DETR based on RT-DETR, which reduces model complexity through deep–shallow feature fusion and backbone optimization, while enhancing contextual information with a re-weighted downsampling mechanism. Zhang et al. Zhou et al. (2022) designed a multi-scale ship detection network using a Cross Stage Partial network with Modified Res2Net and a Feature Pyramid Network with Fusion Coefficients for adaptive multi-scale feature fusion. However, such approaches remain computationally expensive.

To overcome this, the YOLO series Zheng et al. (2022); Cheng et al. (2024) adopts end-to-end design, lightweight backbones, and efficient feature fusion to improve accuracy while reducing computation, becoming the mainstream solution. Nevertheless, most existing methods focus on detection accuracy while neglecting data security. This motivates research on integrating encryption mechanisms into detection frameworks for secure and efficient disease recognition in IoT-based agriculture. Therefore, this paper proposes an integrated framework that combines multi-image encryption with lightweight disease detection, ensuring data security while achieving efficient and robust recognition.

A summary of existing methods, their limitations, and the motivation of this study is provided in **Table 1**.

**Table 1:** Summary of existing methods, limitations, and the proposed solution.

Method	Main focus	Limitation	Our solution
Traditional encryption methods	Data security	Limited consideration of image-specific characteristics and downstream vision tasks	Lightweight image encryption scheme for agricultural image protection
Existing image encryption methods	Encryption performance enhancement	Mainly focus on security evaluation while ignoring recognition requirements	Privacy-preserving framework integrating encryption and disease recognition
Lightweight detection methods	Efficient disease detection	Privacy protection is rarely considered during image transmission	Joint encryption and lightweight detection framework

Although lightweight detectors have achieved significant progress in improving inference efficiency, existing approaches mainly optimize the recognition stage independently and rarely consider the privacy risks caused by image transmission in agriculturalIoT environments. In practical agricultural applications, visual data are collected by distributed devices and transmitted through potentially insecure networks, requiring both efficient disease recognition and reliable data protection. Therefore, a unified framework that combines lightweight detection with image encryption is still lacking. To address this limitation, this study develops a privacy-preserving crop disease recognition framework by integrating multi-image encryption with the lightweight Mamba-LSDet network, achieving a balance between data security and recognition efficiency.

## 3 Multi-image encryption scheme

In this section, the proposed multi-image encryption scheme is introduced to ensure the security and privacy protection of agricultural images during transmission. The encryption scheme is designed to provide strong confidentiality while maintaining computational efficiency for subsequent disease recognition tasks. The overall encryption process consists of five main stages, including key generation, chaotic sequence construction, row–column transformation, DNA-based global diffusion, and ciphertext storage. The following subsections describe the key generation strategy, chaotic sequence construction, pixel permutation, diffusion mechanism, and ciphertext generation process in detail. The overall encryption process is illustrated in **Figure 2**.

**Figure 2:**
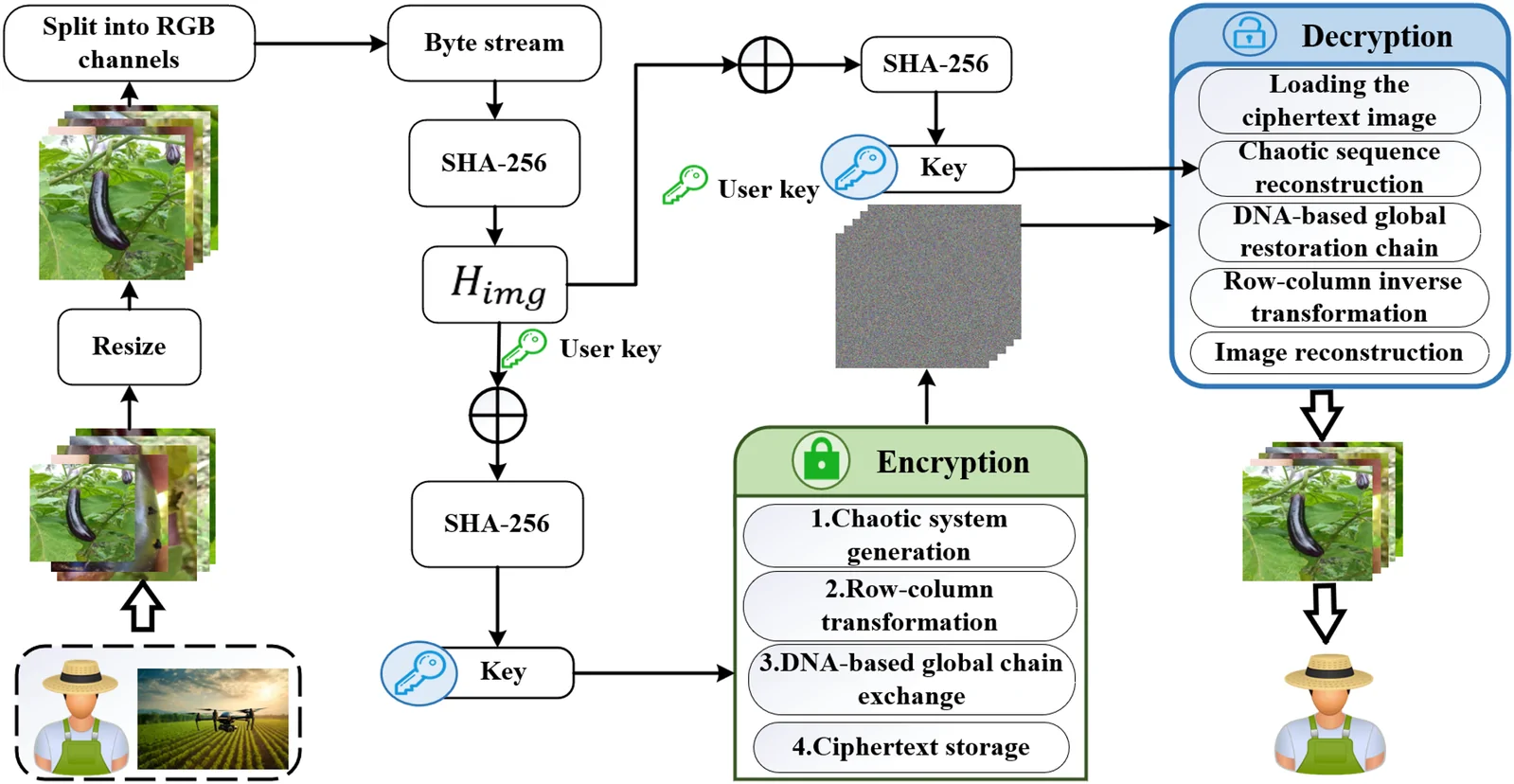
Encryption flowchart.

### 3.1 Key generation

First, all color images to be encrypted, denoted as $\left\{I^{\left(m\right)}\right\}$, are preprocessed by resizing each image to a fixed size *H* × *W* to ensure consistent input length. Each image is split into three channels and read in the standard RGB format. The pixel values of each image are then flattened by channel order and concatenated sequentially to form a continuous RGB byte stream. The 256-bit user key *K_u_* is represented as a 64-digit hexadecimal string and converted into a byte array. Next, the SHA-256 hash of the preprocessed image byte stream is calculated according to Equation 1.

(1) $$H_{img}=\text{SHA}-256\left(\text{bytes}\left(\left\{I^{\left(m\right)}\right\}\right)\right).$$

The user key and the image hash are combined through a bitwise XOR operation, as defined in Equation 2.

(2) $$K_{xor}=K_{u}⊕H_{img}.$$

Finally, another SHA-256 operation is performed on the XOR result to generate the final master key, as expressed in Equation 3.

(3) $$K_{final}=\text{SHA}-256\left(K_{xor}\right).$$

By tightly binding the user-defined key with the normalized image content, *K*_final_ becomes highly sensitive to even a single-bit change in either the key or the image, thereby significantly enhancing the overall security of the encryption system.

### 3.2 Chaotic system generation

The four-dimensional discrete coupled chaotic system is defined by the iterative equations in Equation 4. It is used with initial values [0.1,0.2,0.3,0.4] and parameters (a,b,c,d,t)=(4,4,3.5,2,4). These parameters are empirically selected to ensure chaotic behavior and generate highly random sequences for image encryption, following the parameter selection strategy commonly adopted in chaos-based image encryption studies Tong et al. (2015). The system is iterated for 5 × 10^5^ steps to generate the state matrix $state\in {\mathbb{R}}^{4\times \left(5\times 10^{5}+1\right)}$:

(4) $$\begin{array}{l} x_{n+1}=\text{sin}(x_{n})\cdot \text{sin}(y_{n})-a\;\text{sin}(w_{n}),\\ y_{n+1}=b\;\text{sin}(x_{n})\cdot \text{cos}(y_{n})-x_{n},\\ z_{n+1}=c\;y_{n}+t\;\text{sin}(z_{n}),\\ w_{n+1}=d\;\text{sin}(y_{n})+\text{sin}(w_{n}). \end{array}$$

To eliminate transient effects, a fixed offset *n*_0_ = 1000 is applied, meaning the first 1000 iterations are discarded.

### 3.3 Row–column transformation

Let{*x*[*n*]*,y*[*n*]*,z*[*n*]*,w*[*n*]}*_n_*_≥0_ denote the generated four-dimensional chaotic sequence obtained from the chaotic system, and set the offset parameter to *n*_0_ = 1000. For each color channel (in the order *R* → *G* → *B*), four operations are performed sequentially: row permutation, column permutation, row-wise XOR, and column-wise XOR.

• Row permutation (top to bottom):

For each row *i*, the row permutation parameters are calculated according to Equation 5.

(5) $$\{\begin{array}{l} \rho_{i}=\left\lfloor \left|y\left[n_{0}+1000+i+A\right]\right|H_{c}\right\rfloor \text{mod }H,\\ \sigma_{i}=\left\lfloor \left|z\left[n_{0}+2000+i+K\right]\right|E\right\rfloor \text{mod }W. \end{array}$$

Swap row *i* with row *ρ_i_*, and then circularly shift row *i* to the right by *σ_i_*pixels.

• Column permutation (left to right):

For each column *j*, the column permutation parameters are calculated according to Equation 6.

(6) $$\{\begin{array}{l} \kappa_{j}=\left\lfloor \left|x\left[n_{0}+1500+j+J\right]\right|L\right\rfloor \text{mod }W\\ \tau_{j}=\left\lfloor \left|w\left[n_{0}+2500+j+B\right]\right|I\right\rfloor \text{mod }H, \end{array}$$

Swap column *j* with column *κ_j_*, and then circularly shift column *j* downward by *τ_j_* pixels.

• Row XOR:

For each row *i*, the row-wise XOR operation is performed according to Equation 7.

(7) $$m_{i}=\left(y\left[n_{0}+1000+i+A\right]\cdot H_{c}\right)\text{mod }256,$$

and XOR each byte in row *i* with *m_i_*.

• Column XOR:

For each column *j*, the column-wise XOR operation is performed according to Equation 8.

(8) $$n_{j}=\left(x\left[n_{0}+1000+j+K\right]\cdot E\right)\text{mod }256,$$

and XOR each byte in column *j* with *n_j_*.

### 3.4 DNA-based global chain exchange

Assume that the current batch contains *N* = 3*M* color channels, and the grayscale matrix sequence ${\left\{X^{\left(t\right)}\right\}}_{t=0}^{N-1}$ has been obtained through the initial chaotic preprocessing. This stage consists of three main steps:

•Bitwise cyclic shift and DNA encoding:

The pixel-level control matrix is generated from the chaotic sequence *w*[*n*] according to Equation 9.

(9) $$K\left(i,j\right)=\left\lfloor 256\cdot w\left(\left(iJ+jL\right)\text{mod }30000+n_{0}\right)\right\rfloor \text{mod }8,$$

where K(i,j)∈{0,…,7} represents the number of left shifts. For each pixel (*i*, *j*), $X^{\left(t\right)}\left(i,j\right)$ is first converted into an 8-bit binary string and cyclically left-shifted by *K*(*i*, *j*) bits. Then, the value of *K*(*i*, *j*) mod 8 determines the corresponding DNA encoding rule. The 8 bits are divided into four 2-bit groups and mapped to bases {*A*, *T*, *C*, *G*}. After all channels are encoded, a DNA matrix $P_{1}\in {\left\{A,T,C,G\right\}}^{H\times W\times 4N}$ is obtained.

•Global chain exchange and bidirectional cyclic shift:

The four *N* bases of each pixel in *P*_1_ are flattened into a single chain, resulting in *HW* DNA chains, each of length *L* = 4*N*. A global exchange index is generated from the chaotic sequence *x*[*n*] as $S\left(i,j\right)=HW\cdot x\left(\left(iH_{c}+Ij\right)\text{mod }30000+n_{0}\right)\text{mod }HW.$Two chains (*p,q*) are paired according to *S*, and the shift amount is calculated as *r* = (*S_p_*⊕ *S_q_*) mod *L* modL. Each pair of chains is cyclically shifted by +*r* and −*r*, respectively, and then swapped. Because the operations are inverse and performed once, they can be symmetrically reversed during decryption. After this step, matrix *P*_2_ is obtained.

•Fixed-table decoding:

A decoding table is selected based on *I* mod 8 Every four bases in *P*_2_ are inversely mapped into 8 bits, which are then recombined into pixel values to form the encrypted grayscale stack $Y\in {\mathbb{Z}}_{8}^{H\times W\times N}$. Finally, the encrypted image is reconstructed by merging the (*R,G,B*) channels.

This process employs two cyclic shift operations (an 8-bit shift during encoding and a pairwise +*r/*− *r* inverse shift during exchange) along with chaos-controlled chain swapping. These operations significantly enhance pixel-level bit diffusion and global coupling.

### 3.5 Ciphertext storage

The three channels are recombined in order into a single RGB ciphertext image and saved sequentially as PNG files. Two auxiliary parameter files are also stored, recording the subkey information and the channel start markers. During recovery, the decryption side must use the same chaotic system equations, parameters and initial conditions, the fixed offset, the DNA encoding rule set, and the two parameter files mentioned above.

### 3.6 Decryption process

The decryption process performs the corresponding inverse operations of the encryption procedures. First, loading the ciphertext image: the encrypted RGB image is read and separated into the *R*, *G*, and *B* channels. Next, chaotic sequence reconstruction: using the same preprocessing and SHA-256 calculation as in the encryption stage, the master key *K_final_* is rebuilt, and identical chaotic sequences and subkey parameters are generated. Then, DNA-based global chain restoration: the recorded control indices are applied to perform the inverse operations of global chain exchange and bidirectional cyclic shifts, and the DNA bases are decoded back into pixel bits according to the same rules used in encryption. After that, row–column inverse transformation: the inverse operations of column XOR, row XOR, column permutation, and row permutation are applied sequentially to restore the original pixel arrangement. Finally, image reconstruction: the decrypted channels are combined in RGB order to obtain the plaintext image identical to the original input.

## 4 Mamba-based lightweight shared detection network

To achieve efficient and accurate crop disease identification, a Mamba-based Lightweight Shared Detection Network (Mamba-LSDet) is proposed in this section. The proposed network aims to improve detection accuracy while reducing computational complexity for resource-constrained agricultural monitoring devices. The overall architecture consists of three main components: the MambaOut Cross-Stage Gated Module (MCGM) for feature extraction, the Lightweight Shared Convolutional Detection Head (LSCD) for efficient prediction, and the optimization strategy based on Distribution Focal Loss (DFL). The following subsections introduce the feature extraction module, detection head design, and loss function in detail. The overall architecture of the proposed model is illustrated in **Figure 3**.

**Figure 3:**
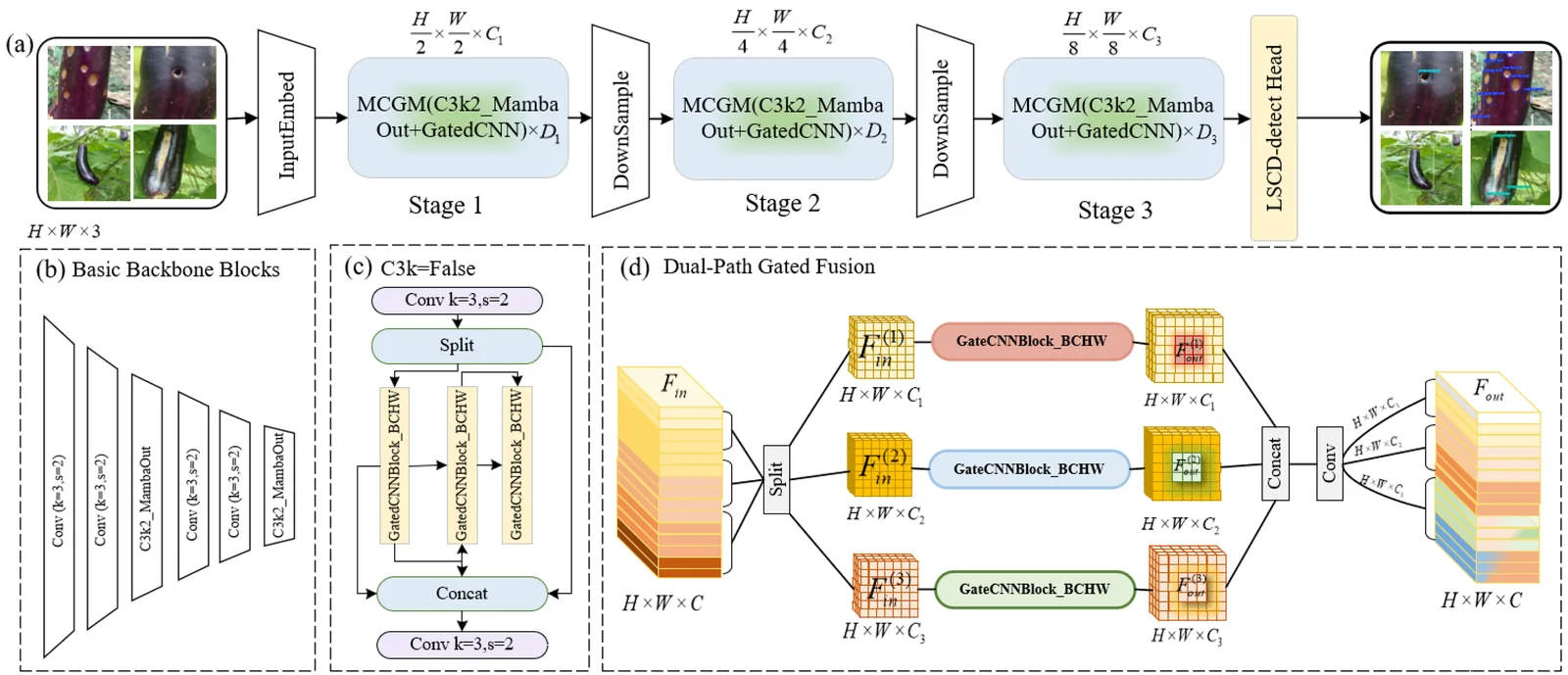
Architecture of the proposed detection model. **(A)** Overall architecture of Mamba-LSDet; **(B)** Basic backbone blocks; **(C)** C3k2 structure with gated convolution blocks; **(D)** Dual-path gated fusion module.

### 4.1 Model overview

In crop disease detection tasks, the efficiency and complexity of the model directly affect its deployment and application in real agricultural environments. Some existing models are too complex, making them unsuitable for edge devices with limited computational resources. Therefore, designing an efficient network architecture is essential. To address this challenge, a Mamba-based Lightweight Shared Detection Network is proposed. As illustrated in **Figure 3A**, the overall architecture of the proposed Mamba-LSDet consists of a multi-stage feature extraction backbone and a lightweight detection head, which jointly achieve efficient crop disease recognition.

In the feature extraction stage, a MambaOut Cross-Stage Gated Module (MCGM) is designed, where gated convolutional units (Gated CNN Blocks) replace the conventional bottleneck structure. By introducing the gating mechanism, the model gains stronger adaptability and selectivity in feature filtering and transmission, significantly improving the representation of disease features. In the detection head, a Lightweight Shared Convolutional Detection Head (LSCD) is proposed, which effectively reduces redundant parameters through the combination of shared convolution towers and hierarchical adapters. Additionally, Distribution Focal Loss (DFL) and an anchor-free decoding strategy are integrated to achieve efficient and stable bounding box regression and classification prediction.

### 4.2 MambaOut cross-stage gated module

In static image-based disease detection tasks, long-range dependency modeling and autoregressive state modeling are not primary requirements.Therefore, the sequence state modeling (SSM) mechanism of Mamba is not directly adopted. Instead, a MambaOut Cross-Stage Gated Module (MCGM) based on the C3k2_MambaOut structure is constructed. As shown in **Figures 3B–D**, the detailed backbone blocks and dual-path gated fusion structure of MCGM are presented, where gated convolution units are introduced to enhance feature selection and cross-stage feature reuse. This module embeds a gated convolution unit (GatedCNN) into the traditional C3k2 framework to achieve dynamic feature selection and cross-stage reuse. This design not only reduces computational overhead but also significantly improves the model’s robustness under low-contrast lesions and complex background conditions.

Assuming the input feature map is $X\in {\mathbb{R}}^{H\times W\times C}$, where *H*, *W*, and *C* denote the height, width, and number of channels, respectively, the gated convolution operation is defined in Equation 10.

(10) $$Y=\sigma \left(W_{g}*X\right)⊙\left(W_{f}*X\right),$$

where *W_g_* and *W_f_* represent the gating and feature convolution kernels, σ is the Sigmoid activation function, and ⊙ denotes element-wise multiplication.

The proposed MCGM module is formulated as shown in Equation 11.

(11) $$\text{MCGM}\left(X\right)=\text{CSP}\left(X\right)+\sum_{i=1}^{n}\text{GatedCNN}\left(X\right),$$

where Skip(·) denotes cross-stage partial connections, and Conv*_g_* denotes the gated convolution operation.

After multi-stage feature extraction and cross-stage gated modeling, three feature vectors at different scales are obtained according to Equation 12.

(12) $$\left\{F_{3},F_{4},F_{5}\right\}=\text{MCGM}\left(X\right),$$

where *F*_3_, *F*_4_, and *F*_5_ correspond to the feature maps from layers P3, P4, and P5, respectively, which serve as the inputs for the subsequent detection head.

### 4.3 Lightweight shared convolutional detection head

In object detection tasks, the detection head usually accounts for a large proportion of the model’s parameters and computational cost, especially when multi-scale feature maps (e.g., *P*_3_, *P*_4_, *P*_5_) are modeled independently, resulting in significant redundancy. To improve detection efficiency, a Lightweight Shared Convolutional Detection Head (LSCD) is designed, which reduces repeated computation through cross-scale shared convolution while maintaining prediction flexibility using learnable scale factors. As illustrated in **Figure 3A**, the proposed LSCD is connected after multi-scale feature extraction and is responsible for generating the final classification and localization predictions.

First, each scale feature *F_i_* is processed by a 3×3 convolution followed by Group Normalization (GN), as described in Equation 13.

(13) $$F_{i}^{\prime }=GN\left(Conv_{3\times 3}^{\left(i\right)}\left(F_{i}\right)\right),$$

where $Conv_{3\times 3}^{\left(i\right)}$ denotes the convolution kernel for the *i*-th scale. The Group Normalization operation is calculated according to Equation 14.

(14) $$\text{GN}\left(x\right)=\gamma \cdot \frac{x-\mu_{G}}{\sqrt{\sigma_{G}^{2}+\varepsilon }}+\beta ,$$

where *μ_G_* and $\sigma_{G}^{2}$ represent the mean and variance of each group, and γ and *β* are learnable scaling and shifting parameters. Compared to BatchNorm, GN is more stable and does not depend on batch size, making it suitable for small-batch agricultural detection tasks.

Next, all $F_{i}^{\prime }$ are fed into a cross-scale shared convolution module (SharedConv) composed of depthwise separable convolution and pointwise convolution, as defined in Equation 15.

(15) $$\hat{F}i=\text{SharedConv}\left(F_{i}^{\prime }\right),$$

where shared weights ensure consistent modeling across different scales and avoid parameter redundancy. Then, ${\hat{F}}_{i}$ is fed into two prediction branches: the box branch predicts the bounding box distribution, and the classification branch predicts class probabilities, as defined in Equation 16.

(16) $$Box_{i}=s_{i}\cdot Conv_{box}\left(\hat{F_{i}}\right),\;Cls_{i}=Conv_{cls}\left(\hat{F_{i}}\right),$$

where *s_i_* is a learnable scale factor that enhances the model’s adaptability to different feature resolutions. The final detection output is defined in Equation 17.

(17) $$O_{i}=\left[Box_{i},Cls_{i}\right].$$

To improve the precision of bounding box regression, Distribution Focal Loss (DFL) is incorporated into the box branch. Specifically, each boundary q∈l,t,r,b is discretized into *R* bins, and the probability distribution is predicted according to Equation 18.

(18) $$p_{k}^{\left(q\right)}=softmax\left(z_{k}^{\left(q\right)}\right),\;k=0,\ldots ,R-1,$$

The boundary position is decoded by expectation according to Equation 19.

(19) $$d_{q}=\sum_{k=0}^{R-1}p_{k}^{\left(q\right)}\cdot k.$$

Finally, the predicted bounding box set is obtained by combining it with the anchor positions *A*, as expressed in Equation 20.

(20) B=dist2bbox(d,A).

By integrating shared convolution, Group Normalization, and DFL-based decoding, the proposed detection head significantly improves detection accuracy and generalization ability while maintaining a lightweight design.

## 5 Performance analysis

This section presents experiments from two perspectives: image encryption and disease recognition. Five color images of eggplant leaf diseases with a resolution of 640×640 are selected as test samples. In the encryption experiments, key sensitivity was evaluated using the Unified Average Changing Intensity (UACI) and the Number of Pixels Change Rate (NPCR) metrics, while statistical characteristics and security performance were analyzed through histogram distribution, pixel correlation, information entropy, PSNR, differential attack analysis (UACI/NPCR), and efficiency comparisons with existing methods. In the recognition experiments, performance evaluation, training analysis, ablation studies, and model comparisons were conducted to validate the effectiveness and robustness of the proposed approach.

### 5.1 Key sensitivity analysis

Key sensitivity is a crucial metric for assessing the security and reliability of an image encryption algorithm. A secure scheme should generate completely different ciphertext images even when the secret key is altered by an extremely small amount. In this study, the NPCR and the UACI are employed as the primary evaluation metrics.

Ideally, the theoretical values of NPCR and UACI are approximately 99.6094% and 33.4635%, respectively. The closer the measured results are to these theoretical values, the higher the key sensitivity and security of the encryption scheme. The NPCR metric is defined in Equation 21.

(21) $$NPCR=\frac{\sum_{i,j}D\left(i,j\right)}{M\times N}\times 100\%,$$

where *M* and *N* denote the height and width of the image, and *D*(*i*, *j*) is defined in Equation 22

(22) $$D\left(i,j\right)=\{\begin{array}{ll} 0, & C_{1}\left(i,j\right)=C_{2}\left(i,j\right)\\ 1, & C_{1}\left(i,j\right)\neq C_{2}\left(i,j\right). \end{array}$$

The UACI metric is calculated according to Equation 23.

(23) $$UACI=\frac{1}{M\times N}\underset{i,j}{\sum }\frac{\left|C_{1}\left(i,j\right)-C_{2}\left(i,j\right)\right|}{255}\times 100\%,$$

where *C*_1_ and *C*_2_ are two ciphertext images generated from the same plaintext when the encryption key differs by only 10^−15^.

Encryption was performed on the three color channels of five test images with a slight key perturbation. The NPCR and UACI results under differential attacks are presented in **Table 2**. The obtained values remain consistently close to the theoretical values, with NPCR around 99.61% and UACI around 33.46%. These results demonstrate the excellent key sensitivity of the proposed algorithm and its strong resistance to differential attacks.

**Table 2:** Key sensitivity analysis.

Image	UACI (%)			NPCR (%)		
	R	G	B	R	G	B
640×640 (1)	33.4603	33.5091	33.4707	99.6040	99.6072	99.6049
640×640 (2)	33.4421	33.5069	33.4390	99.6133	99.5972	99.6103
640×640 (3)	33.4728	33.4246	33.5191	99.6132	99.6248	99.6103
640×640 (4)	33.5115	33.4903	33.4445	99.5988	99.6013	99.6173
640×640 (5)	33.3892	33.4582	33.5086	99.6045	99.6079	99.6057

The excellent NPCR and UACI performance is mainly attributed to the combination of chaotic sequence generation, pixel permutation, and DNA-based diffusion operations. These mechanisms enhance pixel randomness and increase the sensitivity of the ciphertextto slight changes in the plaintext, thereby improving the resistance against differential attacks.

### 5.2 Histogram analysis

The histogram is a useful tool for analyzing the pixel intensity distribution of each color channel in an image. If the histograms of the encrypted image in the R, G, and B channels are close to a uniform distribution, it indicates that the encryption algorithm effectively disrupts the original statistical features. As shown in **Figure 4**, the plaintext image exhibits concentrated pixel distribution with significant fluctuations, whereas the encrypted image shows a flat and uniform distribution. These results confirm that the proposed algorithm effectively conceals grayscale features and statistical information, thereby enhancing ciphertext security.

**Figure 4:**
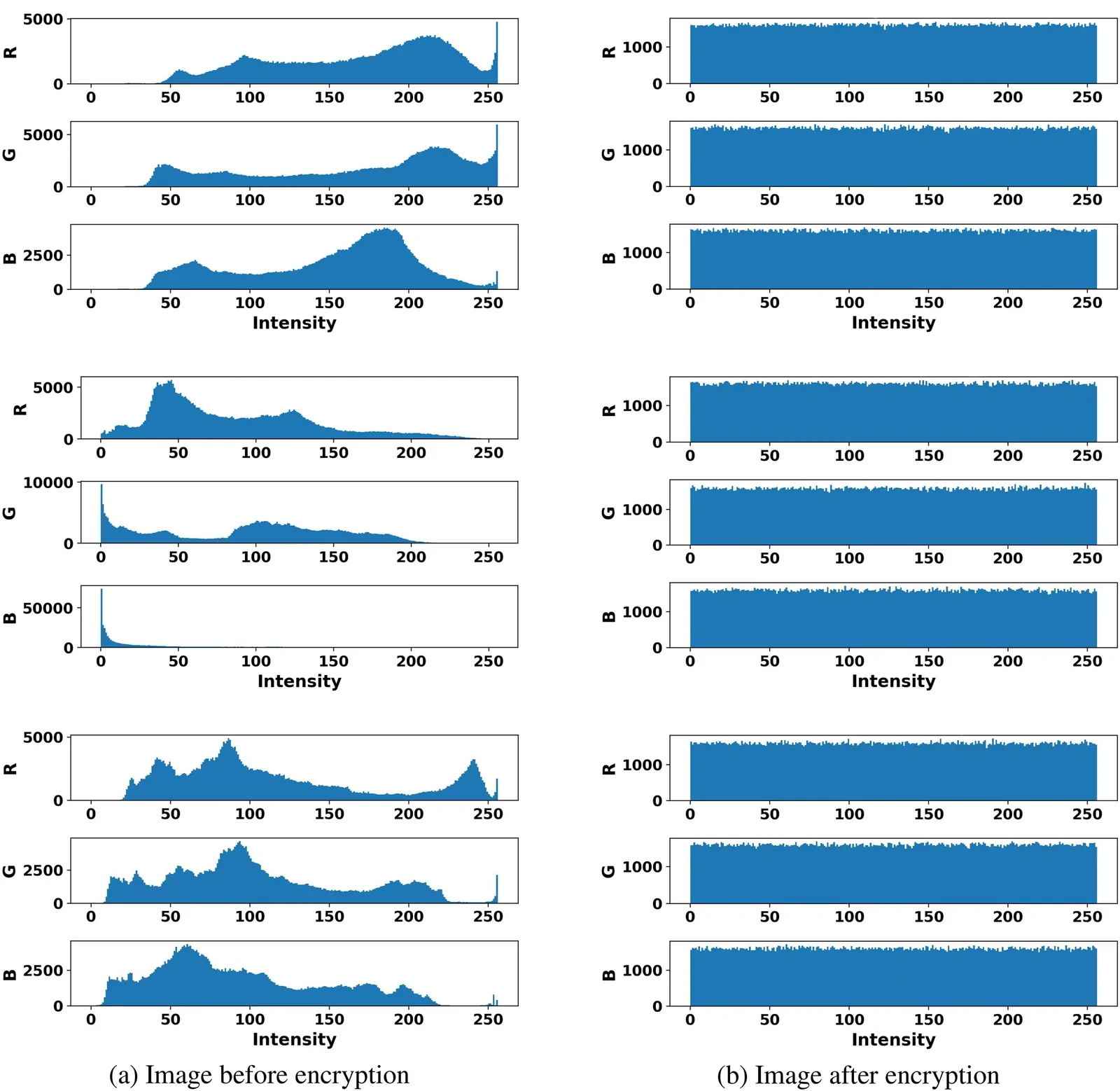
Histograms of different channels before and after encryption.

### 5.3 Correlation analysis

Correlation analysis measures the relationship between adjacent pixels, reflects the algorithm’s ability to reduce redundancy, and shows how well it breaks pixel dependencies.In plaintext images, adjacent pixels are highly correlated, with a correlation coefficient close to 1. In contrast, a well-encrypted image should exhibit correlations close to 0, indicating that the encryption process effectively disrupts spatial relationships and enhances security.

The statistical characteristics of encrypted images are evaluated using the mean and variance, which are defined in Equations 24 and 25.

(24) $$E\left(x\right)=\frac{1}{M\times N}\sum_{i=1}^{M\times N}x_{i},$$

(25) $$D\left(x\right)=\frac{1}{M\times N}\sum_{i=1}^{M\times N}{\left[x_{i}-E\left(x\right)\right]}^{2},$$

where *M* and *N* denote the height and width of the image, and *x_i_* is the gray value of the *i*-th pixel.

The covariance of adjacent pixels is calculated according to Equation 26.

(26) $$\text{cov}\left(x,y\right)=\frac{1}{M\times N}\sum_{i=1}^{M\times N}\left[x_{i}-E\left(x\right)\right]\left[y_{i}-E\left(y\right)\right],$$

The correlation coefficient is then calculated according to Equation 27.

(27) $$r_{xy}=\frac{cov\left(x,y\right)}{\sqrt{D\left(x\right)\cdot D\left(y\right)}},$$

where *x_i_* and *y_i_* represent the values of adjacent pixels in the horizontal, vertical, or diagonal directions.

The correlation coefficients of the R, G, and B channels were calculated in three directions before and after encryption. As shown in **Figure 5**, plaintext images exhibit strong pixel correlations, and the specific numerical results ofthe correlation coefficients are summarized in **Table 3**. The encrypted images show coefficients close to zero, demonstrating that the proposed encryption scheme effectively eliminates spatial correlations and significantly improves statistical security.

**Figure 5:**
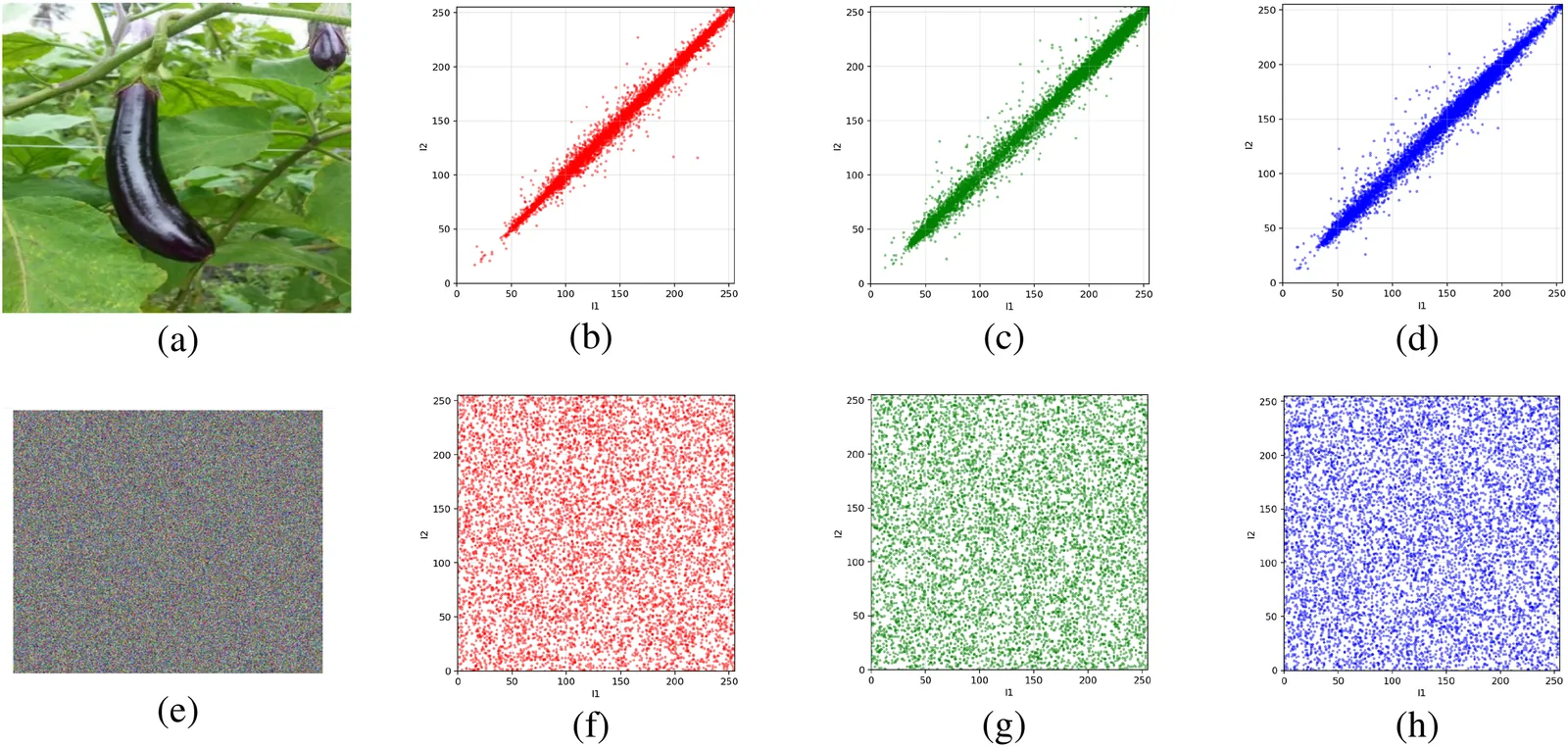
Correlation of adjacent pixels before and after encryption. **(A)** Original image; **(B–D)** Horizontal, vertical, and diagonal adjacency of R, G, and B channels in the original image; **(E)** Encrypted image; **(F–H)** Horizontal, vertical, and diagonal adjacency of R, G, and B channels in the encrypted image.

**Table 3:** Correlation coefficients of original and encrypted images in different directions.

Image	H		V		D	
	Original	Encrypted	Original	Encrypted	Original	Encrypted
640×640 (3)	0.99827	−0.00136	0.99827	−0.00164	0.99653	0.00083
640×640 (4)	0.99888	0.00179	0.99888	0.00219	0.99797	0.00263
640×640 (5)	0.99279	0.00056	0.99279	0.00256	0.98463	−0.00072

### 5.4 Information entropy

Information entropy is a key metric for evaluating the randomness and security of encrypted images. It reflects the uncertainty of pixel distribution by computing the probability distribution of each gray level. For an 8-bit color image with gray values ranging from 0 to 255, the theoretical maximum entropy of each channel is 8 (Shannon, 1948). The information entropy is calculated according to Equation 28.

(28) $$H\left(s\right)=-\sum_{i=1}^{n}P\left(s_{i}\right)\log_{2}\left[P\left(s_{i}\right)\right],$$

where *s_i_* represents the *i*-th gray level, and *P*(*s_i_*) denotes the probability of its occurrence.

The information entropy values of the R, G, and B channels before and after encryption are presented in **Table 4**. It can be observed that the entropy values of the encrypted images are very close to the ideal value of 8, demonstrating that the proposed algorithm significantly improves the randomness of pixel distribution and enhances resistance against statistical analysis attacks.

**Table 4:** Information entropy of images before and after encryption.

Image	Original Image			Encrypted Image		
	R	G	B	R	G	B
640×640(1)	7.59806	7.65883	7.55076	7.99963	7.99953	7.99960
640×640(2)	7.49944	7.51773	5.92704	7.99956	7.99955	7.99958
640×640(3)	7.61108	7.64515	7.60187	7.99955	7.99956	7.99955
640×640(4)	6.94711	7.18877	6.91902	7.99954	7.99956	7.99955
640×640(5)	7.32187	7.08455	7.26476	7.99955	7.99948	7.99956

Peak signal-to-noise ratio (PSNR) is an important metric used to quantitatively evaluate the similarity between the decrypted image and the original image. It is calculated according to Equation 29.

(29) $$PSNR=10\log_{10}\left(\frac{MAX_{I}^{2}}{MSE}\right),$$

where *MAX_I_* represents the maximum pixel intensity value and is set to 255 for 8-bit images. The mean squared error (MSE) is calculated according to Equation 30.

(30) $$MSE=\frac{1}{M\times N}\sum_{i=1}^{M}\sum_{j=1}^{N}{\left(I\left(i,j\right)-I^{\prime }\left(i,j\right)\right)}^{2}.$$

A higher PSNR value indicates that the decrypted image is closer to the original image and that the reconstruction quality is better. Generally, a PSNR value exceeding 30 dB indicates good visual similarity between the decrypted image and the original image Li et al. (2019). The PSNR values of the decrypted images in the R, G, and B channels are presented in **Table 5**. The results demonstrate that the proposed algorithm achieves high-quality image reconstruction after decryption.

**Table 5:** PSNR values of decrypted images.

Image	PSNR (dB)	Image	PSNR (dB)
640 × 640(1)	56.2475	640 × 640(4)	56.7035
640 × 640(2)	55.3953	640 × 640(5)	57.3051
640 × 640(3)	56.4450	–	–

### 5.6 Differential attack resistance analysis

Differential attacks are used to evaluate the sensitivity of an encryption algorithm to small changes in the plaintext. A secure image encryption scheme should generate a completely different ciphertext even when only a single pixel in the original image is modified. Two widely used quantitative metrics for this purpose are the NPCR and the UACI, which are defined in Equations 31–33.

(31) $$NPCR=\frac{\sum_{i,j}D\left(i,j\right)}{M\times N}\times 100\%,$$

(32) $$D\left(i,j\right)=\{\begin{array}{ll} 0, & C_{1}\left(i,j\right)=C_{2}\left(i,j\right),\left(32\right)\\ 1, & C_{1}\left(i,j\right)\neq C_{2}\left(i,j\right), \end{array}$$

(33) $$UACI=\frac{1}{M\times N}\underset{i,j}{\sum }\frac{\left|C_{1}\left(i,j\right)-C_{2}\left(i,j\right)\right|}{255}\times 100\%,$$

where *C*_1_ and *C*_2_ denote the ciphertext images generated from plaintexts differing by only one pixel, M and N represent the image dimensions.

In the experiments, a single pixel in the original image was randomly modified and the corresponding image was re-encrypted. The NPCR and UACI values for the R, G, and B channels were then calculated. The comparison results of NPCR and UACI underdifferent encryption methods are presented in **Table 6**. The NPCR values are close to the ideal value of 99.6%, and the UACI values are close to 33%. These results indicate that the proposed encryption algorithm is highly sensitive to minor changes in the plaintext and exhibits strong resistance against differential attacks.

**Table 6:** Results of differential attack experiments (NPCR and UACI).

Image	NPCR (%) (R/G/B)			UACI (%) (R/G/B)		
1	99.6233	99.5928	99.6069	33.5012	33.4525	33.4301
2	99.5886	99.6218	99.6084	33.4236	33.4566	33.4847
3	99.6038	99.6074	99.6113	33.4911	33.4281	33.4041
4	99.6132	99.6093	99.5976	33.4578	33.5129	33.4601
5	99.6055	99.6189	99.6018	33.3964	33.4435	33.4944

### 5.7 Scheme comparison

This section presents a comparison of the proposed encryption scheme (denoted as our scheme) with several representative methods described in the literature Zhang et al. (2024a); Huang and Gao (2024); Teng et al. (2024) in terms of encryption efficiency. All programs were implemented on a personal computer equipped with a 13th Generation Intel^®^ Core™ i7-13700H processor (2.40 GHz) and 16 GB of RAM, and the programming environment was Python 3.13.4.

To evaluate the efficiency of the proposed approach, three representative multi-image encryption methods were selected for comparison. Gao and Teng (2025) (GT for short) propose a two-dimensional chaotic map based multi-image joint encryptionmethod that secures multi-size images via three-dimensional mapping, permutation, and diffusion. Huang et al. Huang and Gao (2024) (HG for short) designed a method that integrates an improved spatiotemporal chaotic system with fractal geometry to generate stronger chaotic sequences and pixel scrambling strategies. Teng et al. Teng et al. (2024) (TCY+ for short) introduced a dual dynamic coupled chaotic system with delayed feedback, enhancing security through chaotic permutation and diffusion.

The encryption and decryption time comparison with existing methods is presented in **Table 7**. The proposed scheme achieves an average encryption and decryption time of 0.339 s per image, where as the GT Gao and Teng (2025) requires 0.712s, the HG Huang and Gao (2024) requires 0.526s, and the TCY+ []? requires 0.403s. The proposed method is therefore noticeably faster than these representative schemes. Meanwhile, the entropy, NPCR, and UACI values are comparable to or better than those of the competing methods, indicating that the improvement in computational efficiency does not compromise security performance. This demonstrates that the proposed scheme achieves a favorable balance between efficiency and security.

**Table 7:** Encryption efficiency and security performance comparison of different schemes.

Scheme	Image size	Time (s)	Entropy	NPCR (%)	UACI (%)
Our scheme	640×640	**0.339**	7.9996	99.6080	33.4698
GT Gao and Teng (2025)	640×640	0.712	7.9995	99.6093	33.4674
HG Huang and Gao (2024)	640×640	0.526	7.9994	99.5179	33.4516
TCY+ Teng et al. (2024)	640×640	0.403	7.9995	99.6323	33.4363

Bold values denote the optimal results achieved by our proposed method compared with other competing approaches.

In practical agricultural IoT applications, the proposed encryption scheme provides an efficient solution for secure image transmission in large-scale monitoring scenarios. The low computational cost enables deployment on resource-constrained edge devices, where secure data protection and real-time processing are both required.

### 5.8 Detection model performance evaluation

The performance comparison between the baseline and improved models is presented in **Tables 8**, **9**. The results indicate that the improved model significantly reduces complexity while maintaining, and in some cases improving, detection accuracy. At the overall level, the precision (Box(P)) increased from 0.829 to 0.846, while the recall (R) improved from 0.776 to 0.798. Similarly, mAP@50 and mAP@50–95 rose from 0.825 and 0.488 to 0.841 and 0.505, respectively, indicating that the improved model outperforms the baseline even in terms of detection precision.For specific categories, the mAP@50–95 of Fruit Rot and Healthy increased from 0.566 and 0.686 to 0.586 and 0.699, demonstrating enhanced performance in key classes. Moreover, minor but consistent improvements were observed in small-object categories such as Fruit Borer and Melon Thrips, confirming the stability and robustness of the proposed model across diverse detection tasks.

**Table 8:** Detection performance of the baseline YOLOv11n model.

Class	Img	Inst	Box(P)	R	mAP@50	mAP@50-95
all	509	1119	0.829	0.776	0.825	0.488
Fruit_Rot	77	331	0.822	0.845	0.864	0.566
Fruit_borer	161	212	0.878	0.828	0.861	0.379
Healthy	139	277	0.900	0.919	0.953	0.686
Melon_Thrips	125	299	0.740	0.540	0.622	0.320

**Table 9:** Detection performance of the improved model.

Class	Img	Inst	Box(P)	R	mAP@50	mAP@50-95
all	509	1119	0.846	0.798	0.841	0.505
Fruit_Rot	77	331	0.835	0.858	0.883	0.586
Fruit_borer	161	212	0.892	0.848	0.883	0.397
Healthy	139	277	0.907	0.932	0.962	0.699
Melon_Thrips	125	299	0.750	0.553	0.633	0.332

In practical agricultural IoT scenarios, the proposed framework provides secure image transmission and efficient disease recognition for resource-constrained devices. The lightweight encryption scheme reduces the computational burden during image protection, while the lightweight Mamba-LSDet network enables accurate disease identification with reduced model complexity. Therefore, the proposed framework is suitable for large-scale agricultural monitoring systems requiring both data privacy protection and real-time recognition capability.

### 5.9 Training process analysis

The training process was visualized through loss functions and performance metrics, as shown in **Figure 6**. The top row shows the training results, and the bottom row presents the corresponding validation results. As the number of epochs increased, the values of box_loss, cls_loss, and dfl_loss steadily decreased and stabilized, indicating effective convergence in bounding box regression, classification, and distribution fitting. The validation curves exhibited similar trends, with no signs of significant overfitting.

**Figure 6:**
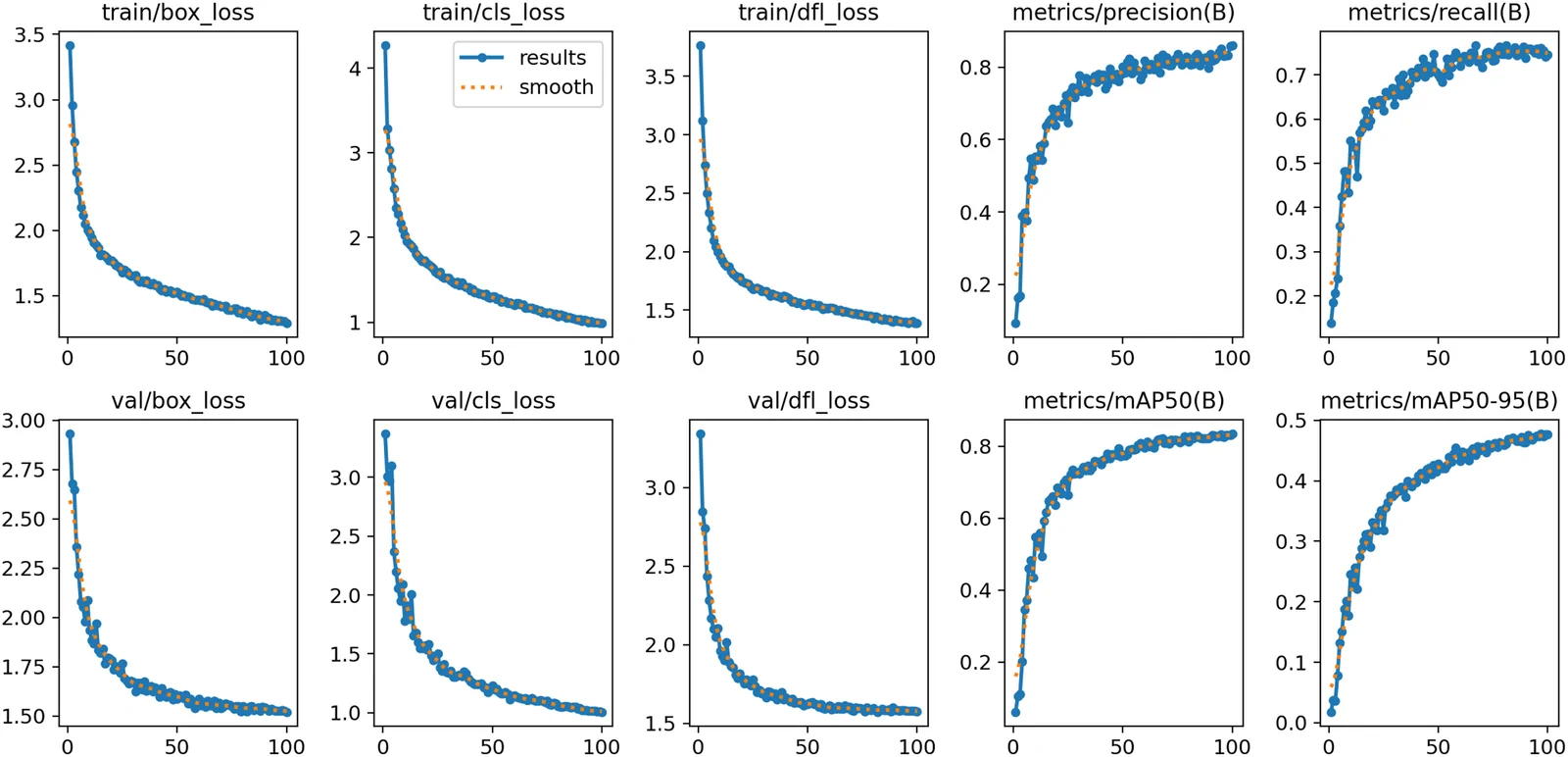
Visualization of the training and validation process. The top row shows the loss and performance metrics on the training set, and the bottom row shows the results on the validation set.

For performance metrics, precision and recall increased steadily, while mAP@50 and mAP@50–95 improved consistently and reached high levels. These results demonstrate that detection accuracy improved progressively and stabilized after about 100 epochs. Overall, the training curves confirm strong model convergence, and the consistency between training and validation performance verifies the effectiveness and reliability of the proposed approach.

### 5.10 Ablation study

The ablation results of the proposed modules are presented in **Table 10**. The results evaluate the contribution of each module to detection accuracy and computational efficiency. When the C3k2_MambaOut or LSCD modules were appliedindividually, performance gains were limited, and slight accuracy drops were occasionally observed. However, combining both modules led to significant improvements. Specifically, the number of parameters was reduced by about 16.0%, and computational complexity (GFLOPs) decreased by approximately 15.6%. Meanwhile, mAP@50 increased from 0.825 to 0.840, and mAP@50–95 improved from 0.488 to 0.505.

**Table 10:** Ablation results of the proposed modules.

Model	C3k2_MambaOut	LSCD	Parameters	GFLOPs	mAP@50	mAP@50–95
1	×	×	2,590,620	6.4	0.825	0.488
2	×	✓	2,426,967	5.7	0.833	0.474
3	✓	×	2,334,254	6.1	0.811	
Ours	✓	✓	2,177,337	5.4	**0.840**	**0.505**

Bold values denote the optimal results achieved by our proposed method compared with other competing approaches.

These results show that the C3k2_MambaOut module enhances feature representation and multi-scale fusion, while the LSCD module reduces redundant computation through shared convolution. Their combination achieves a better balance between accuracy and efficiency, demonstrating strong synergy in lightweight detection network design.

### 5.11 Model comparison

To comprehensively validate the performance of the proposed model, comparisons with several mainstream object detection networks, including Faster R-CNN, SSD, RT-DETR, and lightweight YOLO variants (YOLOv5n, YOLOv6n, YOLOv7n, YOLOv8n, YOLOv9t, YOLOv10n, and YOLOv11n), are presented in **Table 11**. The results demonstrate that the proposed model achieves a favorable balance between detection accuracy and computational efficiency, outperforming most compared methods while maintaininga lightweight design.

**Table 11:** Comparison of detection performance with different models.

Model	Parameters	GFLOPs	mAP@50-95	mAP@50	FPS
Faster-RCNN	314,109,132	341.2	–	–	–
SSD	53,254,411	112.5	–	–	–
RT-DETR	81,648,102	109.6	–	–	–
YOLOv5n	2,503,724	7.1	0.449	0.803	–
YOLOv6n	4,234,140	11.8	0.447	0.790	–
YOLOv7n	5,630,501	13.4	0.426	0.768	–
YOLOv8n	3,011,612	8.2	0.461	0.817	–
YOLOv9t	1,971,564	7.6	0.467	0.810	–
YOLOv10n	2,695,976	8.2	0.445	0.771	–
YOLOv11n (Baseline)	2,590,620	6.4	0.488	0.825	–
Ours	**2,177,337**	**5.4**	**0.505**	**0.840**	–

Bold values denote the optimal results achieved by our proposed method compared with other competing approaches.

Under the same experimental settings, the proposed model achieves a parameter count of 2.18M and a computational cost of 5.4 GFLOPs, both lower than those of YOLOv11n (2.59M and 6.4 GFLOPs). Moreover, mAP@50 improved from 0.825 to 0.840, and mAP@50–95 increased from 0.488 to 0.505. Compared with stateoftheart lightweight detectors such as YOLOv8n and YOLOv9t, our approach still shows superior accuracy. These results demonstrate that the proposed model significantly reduces computational cost while maintaining high detection performance, highlighting its strong generalization ability and deployment potential in real-world scenarios.

## 6 Conclusion

In this study, a unified framework that integrates image encryption with lightweight disease detection was proposed to address the dual challenges of data security and recognition accuracy in agricultural systems. The proposed multi-image encryption scheme, based on a four-dimensional chaotic system and DNA-based global chain operations, achieves strong randomness and high information entropy while maintaining high efficiency. On the detection side, the Mamba-LSDet model incorporates dynamic feature selection and cross-scale feature sharing, delivering high detection performance with reduced computational cost. Experimental results demonstrate that our approach ensures secure transmission and storage of sensitive agricultural data while significantlyimproving detection accuracy compared with existing methods. Future work will focus on further enhancing encryption efficiency, improving the model’s adaptability to different crops and environmental conditions, and deploying the system on real-world agricultural devices to strengthen its practicality and robustness.

## 7 Discussion

This study presents a privacy-preserving crop disease recognition framework that combines 4D DNA chaotic image encryption with the lightweight Mamba-LSDet network for agricultural IoT applications. The proposed encryption scheme achieves strong security performance with near-ideal entropy values, weak pixel correlations, and high resistance against statistical and differential attacks. Meanwhile, Mamba-LSDet provides accurate disease recognition after image decryption and achieves improved detection performance compared with YOLOv11n.

The proposed framework currently follows a sequential pipeline, where images are encrypted before transmission and decrypted before disease recognition. Therefore, it does not achieve true encrypted-domain inference. Future studies will investigate privacy-preserving inference methods, such as homomorphic encryption and secure multi-party computation, to further improve data confidentiality. In addition, although the framework shows promising results, experiments are currently limited to eggplant disease datasets, and further validation on multi-crop datasets is required to evaluate its generalization capability.

Moreover, the encryption and detection modules are optimized independently without joint optimization or explicit trade-off analysis. Standard cryptographic algorithms, such as AES-256-GCM, are designed for general-purpose data protection, whereas this study focuses on developing an image-oriented encryption scheme with a balance between security and computational efficiency. Future work will further explore joint optimization strategies and comparisons with conventional cryptographic baselines.

## Data Availability

The original contributions presented in the study are included in the article/supplementary material. Further inquiries can be directed to the corresponding author.

## References

[B1] Barjaktarovic M. Santoni M. Bruzzone L. (2024). Design and verification of a low-cost multispectral camera for precision agriculture application. IEEE J. Sel. Top. Appl. Earth Obs. Remote Sens. 17, 6945–6957. doi: 10.1109/JSTARS.2024.337710425079929

[B2] Cheng T. Song L. Ge Y. Liu W. Wang X. Shan Y. (2024). Yolo-world: Real-time open-vocabulary object detection 16901–16911. doi: 10.1109/CVPR52733.2024.01599

[B3] Du L. Sun Y. Chen S. Feng J. Zhao Y. Yan Z. . (2022). A novel object detection model based on faster r-cnn for spodoptera frugiperda according to feeding trace of corn leaves. Agriculture 12, 248. doi: 10.3390/agriculture1202024830654563

[B4] Gao J. Teng L. (2025). A chaotic multi-image encryption scheme based on block space jump scrambling and dynamic sliding queue diffusion. Nonlinear. Dyn. doi: 10.1007/s11071-025-11690-330311153

[B5] Gao S. Zhang Z. Iu H. H.-C. Ding S. Mou J. Erkan U. . (2025). A parallel color image encryption algorithm based on a 2-d logistic-rulkov neuron map. IEEE Internet Things. J. 12, 18115–18124. doi: 10.1109/JIOT.2025.354009725079929

[B6] Ghosh A. Kumar A. Biswas G. (2024). Exponential population growth and global food security: challenges and alternatives 1–20. doi: 10.1016/B978-0-443-13993-2.00001-3

[B7] Goudjil A. Benyoucef A. Hamadouche M. Riahla M. A. (2025). Efficient dna-based logistic mapping algorithm for color image encryption. ICST. Trans. Scalable. Inf. Syst. 12. doi: 10.4108/eetsis.6800

[B8] Huang L. Gao H. (2024). Multi-image encryption algorithm based on novel spatiotemporal chaotic system and fractal geometry. IEEE Trans. Circuits. Syst. I.: Regular. Papers. 71, 3726–3739. doi: 10.1109/TCSI.2024.340780925079929

[B9] Iftikhar M. Kandhro I. A. Kausar N. Kehar A. Uddin M. Dandoush A. (2024). Plant disease management: a fine-tuned enhanced cnn approach with mobile app integration for early detection and classification. Artif. Intell. Rev. 57. doi: 10.1007/s10462-024-10809-z30311153

[B10] Jammula M. Vakamulla V. M. Kondoju S. K. (2022). Retracted: Artificial intelligence framework-based ultra-lightweight communication protocol for prediction of attacks in Internet of Things environment. Trans. Emerging. Telecommun. Technol. 34. doi: 10.1002/ett.468042587375

[B11] Li K.-R. Duan L.-J. Deng Y.-J. Liu J.-L. Long C.-F. Zhu X.-H. (2024). Pest detection based on lightweight locality-aware faster r-cnn. Agronomy 14, 2303. doi: 10.3390/agronomy1410230330654563

[B12] Li L. Chang C.-C. Chen H. (2019). An improved bidirectional shift-based reversible data hiding scheme using double-way prediction strategy. Secur. Commun. Netw. 2019, 3031506. doi: 10.1155/2019/3031506

[B13] Li X. Hou B. Zhang R. Liu Y. (2023). A review of rgb image-based internet of things in smart agriculture. IEEE Sens. J. 23, 24107–24122. doi: 10.1109/JSEN.2023.330977425079929

[B14] Liang C. Huang T. Dai Q. Li Z. Yu S. (2024). A single-sized metasurface for image steganography and multi-key information encryption. Engineering 41, 61–70. doi: 10.1016/j.eng.2024.04.01542574925

[B15] Liu Z. Gao A. Sheng H. Wang X. (2025). Identification of diabetic retinopathy lesions in fundus images by integrating cnn and vision mamba models. PloS One 20, e0318264. doi: 10.1371/journal.pone.0318264 39874303 PMC11774367

[B16] Moreira Bezerra J. I. Valduga de Almeida Camargo V. Molter A. (2021). A new efficient permutation-diffusion encryption algorithm based on a chaotic map. Chaos. Solitons. Fractals. 151, 111235. doi: 10.1016/j.chaos.2021.11123542574925

[B17] Ngugi H. N. Ezugwu A. E. Akinyelu A. A. Abualigah L. (2024). Revolutionizing crop disease detection with computational deep learning: a comprehensive review. Environ. Monit. Assess. 196, 302. doi: 10.1007/s10661-024-12454-z 38401024 PMC10894121

[B18] Priyanka Baranwal N. Singh K. N. Singh A. K. . (2024). Yolo-based roi selection for joint encryption and compression of medical images with reconstruction through super-resolution network. Future Gener. Comput. Syst. 150, 1–9. doi: 10.1016/j.future.2023.08.01842574925

[B19] Qian K. Xiao Y. Wei Y. Liu D. Wang Q. Feng W. (2023). A robust memristor-enhanced polynomial hyper-chaotic map and its multi-channel image encryption application. Micromachines 14, 2090. doi: 10.3390/mi14112090 38004947 PMC10673070

[B20] Quach L.-D. Quoc K. N. Quynh A. N. Ngoc H. T. Thai-Nghe N. (2024). Tomato health monitoring system: Tomato classification, detection, and counting system based on yolov8 model with explainable mobilenet models using grad-cam++. IEEE Access 12, 9719–9737. doi: 10.1109/ACCESS.2024.335180525079929

[B21] Rehman M. U. Shafique A. Ghadi Y. Y. Boulila W. Jan S. U. Gadekallu T. R. . (2022). A novel chaos-based privacy-preserving deep learning model for cancer diagnosis. IEEE Trans. Network. Sci. Eng. 9, 4322–4337. doi: 10.1109/TNSE.2022.319923525079929

[B22] Sangaiah A. K. Yu F.-N. Lin Y.-B. Shen W.-C. Sharma A. (2024). Uav t-yolo-rice: An enhanced tiny yolo networks for rice leaves diseases detection in paddy agronomy. IEEE Trans. Network. Sci. Eng. 11, 5201–5216. doi: 10.1109/TNSE.2024.335064025079929

[B23] Shannon C. E. (1948). A mathematical theory of communication. Bell. System. Tech. J. 27, 379–423, 623–656. doi: 10.1002/j.1538-7305.1948.tb01338.x42587375

[B24] Teng L. Cao P. Liu Y. (2024). Multi-image encryption algorithm based on novel spatiotemporal chaotic system and dynamical chaotic trajectories. IEEE Trans. Circuits. Syst. Video. Technol. doi: 10.1109/TCSI.2024.340780925079929

[B25] Tong X.-J. Zhang M. Wang Z. Liu Y. Xu H. Ma J. (2015). A fast encryption algorithm of color image based on four-dimensional chaotic system. J. Visual Commun. Image. Represent. 33, 219–334. doi: 10.1016/j.jvcir.2015.09.01442574925

[B26] Wang L. Chen Z. Sun X. He C. (2024). Region of interest encryption algorithm for images based on lifting scheme and object detection. Cluster. Comput. 28. doi: 10.1007/s10586-024-04746-330311153

[B27] Wang X. Liu P. (2022). A new full chaos coupled mapping lattice and its application in privacy image encryption. IEEE Trans. Circuits. Syst. I.: Regular. Papers. 69, 1291–1301. doi: 10.1109/TCSI.2021.313331825079929

[B28] Wenjuan G. Shuo F. Quan F. Xiangzhou L. Xueze G. (2024). Cotton leaf disease detection method based on improved ssd. Int. J. Agric. Biol. Eng. 17, 211–220. doi: 10.25165/j.ijabe.20241702.8574

[B29] Yang H. Liu J. Mei G. Yang D. Deng X. Duan C. (2023). Research on real-time detection method of rail corrugation based on improved shufflenet v2. Eng. Appl. Artif. Intell. 126, 106825. doi: 10.1016/j.engappai.2023.10682542574925

[B30] Yang S. Li Z. Tan W. (2024). Enhanced image encryption with compression using improved coupled map lattices and fisher–yates permutation–diffusion. Eur. Phys. J. Plus. 139. doi: 10.1140/epjp/s13360-024-05413-7

[B31] Yu W. Wang X. (2024). Mambaout: Do we really need mamba for vision? doi: 10.1109/CVPR52734.2025.00423

[B32] Zhang L. Wang Q. Yu Z. Li J. Bai X. Zhou X. . (2024a). Non-cascaded and crosstalk-free multi-image encryption based on optical scanning holography using 2d orthogonal compressive sensing. IEEE Trans. Image. Process. doi: 10.1109/TIP.2024.3468916 39361468

[B33] Zhang Q. Li H. Shao M. Liang H. (2025). Learned image compression with cnn-localmamba architectures and multi-context entropy model. Cluster. Comput. 28. doi: 10.1007/s10586-025-05133-240102317

[B34] Zhang R. Xu L. Yang S. Wang L. (2024b). Mambareid: Exploiting vision mamba for multi-modal object re-identification. Sensors 24, 4639. doi: 10.3390/s24144639 39066036 PMC11280729

[B35] Zhao H. Wang S. Fu Z. (2024). A new image encryption algorithm based on cubic fractal matrix and l-lccml system. Chaos. Solitons. Fractals. 185, 115076. doi: 10.1016/j.chaos.2024.11507642574925

[B36] Zheng Z. Wang P. Ren D. Liu W. Ye R. Hu Q. . (2022). Enhancing geometric factors in model learning and inference for object detection and instance segmentation. IEEE Trans. Cybern. 52, 8574–8586. doi: 10.1109/TCYB.2021.3095305 34437079

[B37] Zhou D. Xu H. Liu W. Liu F. (2025). Ln-detr: cross-scale feature fusion and re-weighting for lung nodule detection. Sci. Rep. doi: 10.1038/s41598-025-00309-7 40319047 PMC12049520

[B38] Zhou Y. Lu C. Lu Z. Guo Z. Ye C. Tsukruk V. V. . (2023). Chiroptical nanocellulose bio-labels for independent multi-channel optical encryption. Small 19. doi: 10.1002/smll.202303064 37162465

[B39] Zhou K. Zhang M. Wang H. Tan J. (2022). Ship detection in sar images based on multi-scale feature extraction and adaptive feature fusion. Remote Sens. 14. doi: 10.3390/rs14030755

[B40] Zuhair M. Z. Ashraf S. Iqbal M. Rizvi S. Z. R. Khokhar A. Nadeem M. (2023). Broaching uncouth water level snag in underground agriculture field through wireless sensors. Suranaree. J. Sci. Technol. 30, 010248. doi: 10.55766/sujst-2023-04-e0855

